# Supplementary material for: Direct photo-patterning of halide perovskites toward machine-learning-assisted erasable photonic cryptography
Source: Nat Commun. 2025 Apr 7;16:3316. doi: 10.1038/s41467-025-58677-7 (PMC11977006; doi:10.1038/s41467-025-58677-7)
Supplement: Supplementary file 1 — Supplementary Information [file 41467_2025_58677_MOESM1_ESM.pdf]

Supplementary Information

Direct photo-patterning of halide perovskites toward machine-learning-assisted erasable photonic cryptography

Yingjie Zhao[1†], Mengru Zhang[1†], Zhaokai Wang[1], Haoran Li[1], Yi Hao[1], Yu Chen[2], Lei Jiang[3,4,5], Yuchen Wu[3,4,5]*, Shuang-Quan Zang[1,*], and Yanlin Song[1,6]*

[1]College of Chemistry and Pingyuan Laboratory, Zhengzhou University, Zhengzhou 450001, P. R. China

[2]The Institute of High Energy Physics, Chinese Academy of Sciences, Beijing, 100049, P. R. China

[3]Key Laboratory of Bio-inspired Materials and Interfacial Science, Technical Institute of Physics and Chemistry, Chinese Academy of Sciences, Beijing, 100190, P. R. China

[4]Suzhou Institute for Advanced Research, University of Science and Technology of China, Suzhou 215123, China

[5]University of Chinese Academy of Sciences (UCAS), Beijing 100049, China

[6]Key Laboratory of Green Printing, Institute of Chemistry, Chinese Academy of Sciences, Beijing, 100190, P. R. China

†These authors contributed equally to this work.

*Correspondence and requests for materials should be addressed to Y. C. Wu (wuyuchen@iccas.ac.cn), S. Q. Zang (zangsqzg@zzu.edu.cn), and Y. L. Song (ylsong@iccas.ac.cn).

This file includes:

Supplementary Figures 1 to 41

Supplementary Notes 1 to 3

Supplementary Tables 1 to 2

Supplementary References

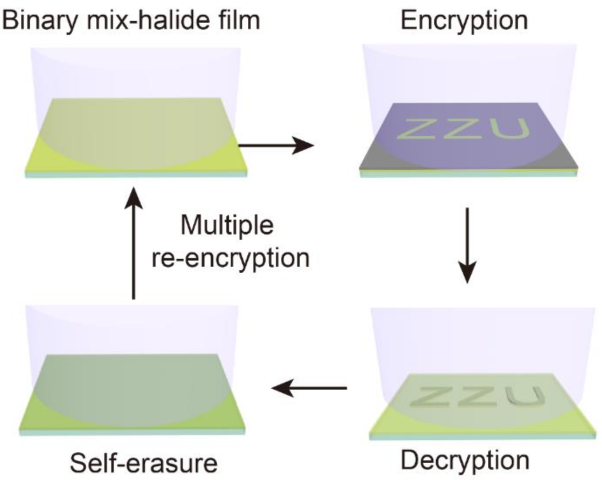

Supplementary Fig. 1. Schematic diagram of direct photo-patterning technique based on binary mix-halide perovskite films.

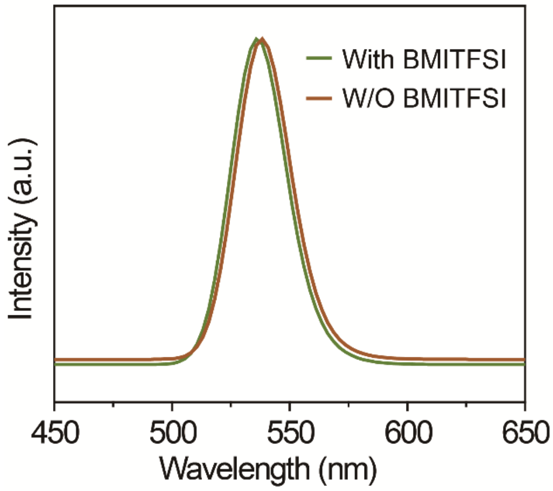

Supplementary Fig. 2. Photoluminescence spectra of perovskite films fabricated without and with BMITFSI additive, exhibiting little spectral difference.

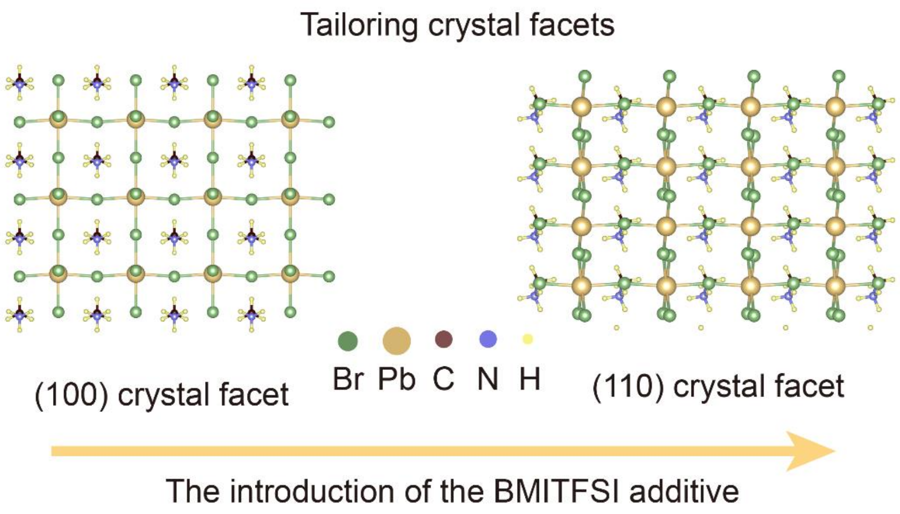

Supplementary Fig. 3. The result of tailoring crystal facets of perovskite with the introduction of the BMITFSI additive.

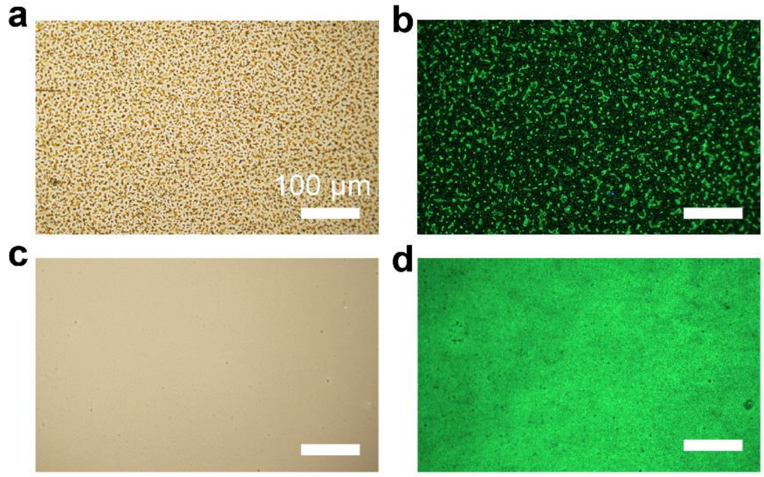

Supplementary Fig. 4. Morphology and photoluminescence photographs of perovskite films fabricated without (a, b) additive and (c, d) with BMITFSI additive. Perovskite films with additives show a denser film morphology with a smaller grain size.

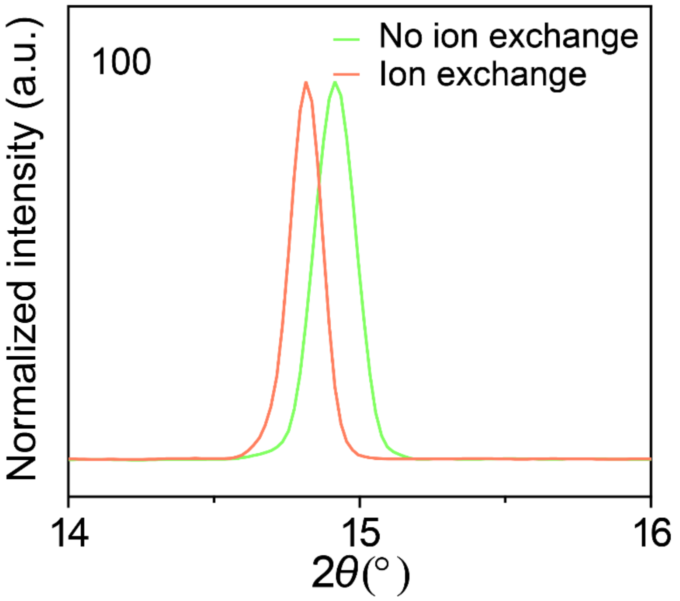

Supplementary Fig. 5. XRD patterns of binary mix-halide perovskite films before and after ion exchange**.**

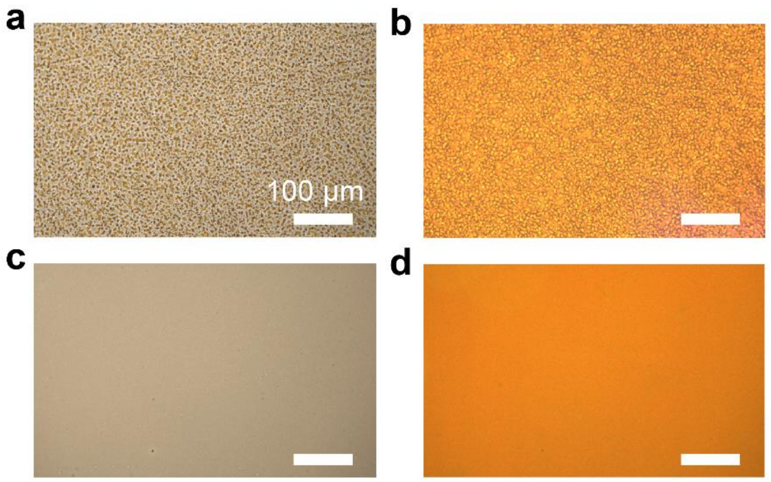

Supplementary Fig. 6. Morphology and photoluminescence photographs of mix-halide perovskite films with MAI concentration of 0.1 mg/ml fabricated without (a, b) BMITFSI additive and (c, d) with BMITFSI additive. Perovskite films with additives exhibit smaller crystal domains and a denser structure.

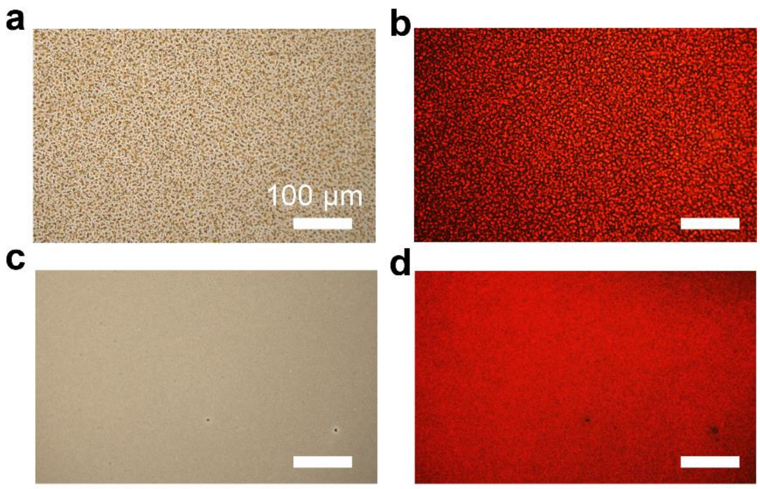

Supplementary Fig. 7. Morphology and photoluminescence photographs of mix-halide perovskite films with MAI concentration of 0.4 mg/ml fabricated without (a, b) BMITFSI additive and (c, d) with BMITFSI additive. Perovskite films with additives exhibit smaller crystal domains and a denser structure.

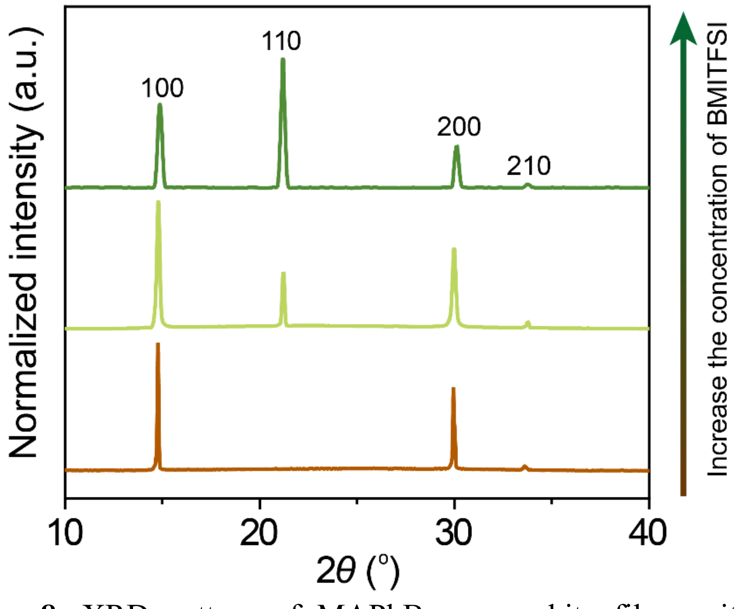

Supplementary Fig. 8. XRD pattern of $MAPbBr_3$ perovskite films with the MAI doping concentration of 0.1 mg/ml under different ratios of BMITFSI additives. The perovskite films present the (110)-dominant crystallographic orientation with the increased proportion of BMITFSI additives.

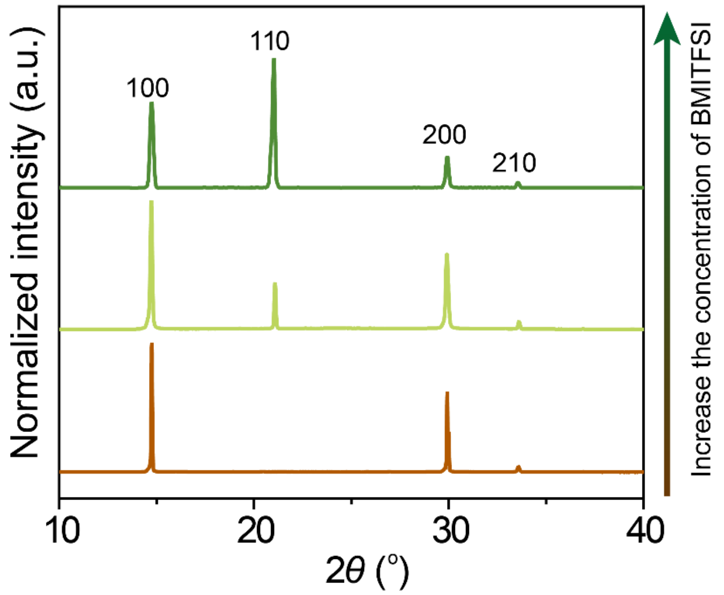

Supplementary Fig. 9. XRD pattern of $MAPbBr_3$ perovskite films with the MAI doping concentration of 0.4 mg/ml under different ratios of BMITFSI additives. The perovskite films present the (110)-dominant crystallographic orientation with the increased proportion of BMITFSI additives.

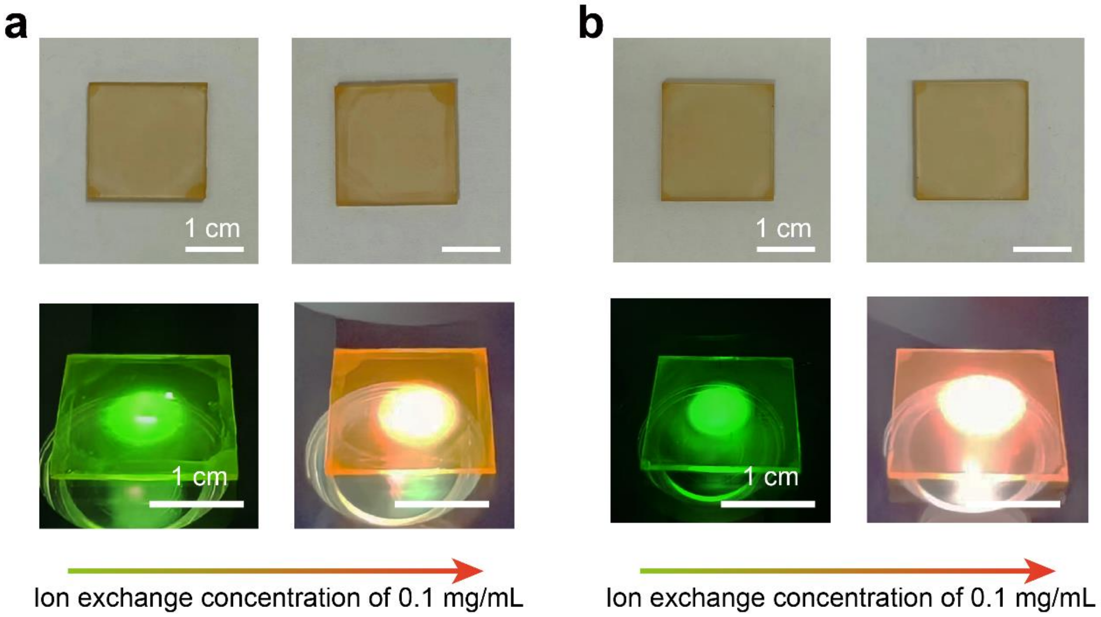

Supplementary Fig. 10. Optical and photoluminescence photographs of perovskite films (a) without additive and (b) with BMITFSI additive after ion exchange with the same concentration of MAI (0.1 mg/mL). Compared to the perovskite films without additives, red-shifted photoluminescence for perovskite films with additives indicates a faster ion exchange rate.

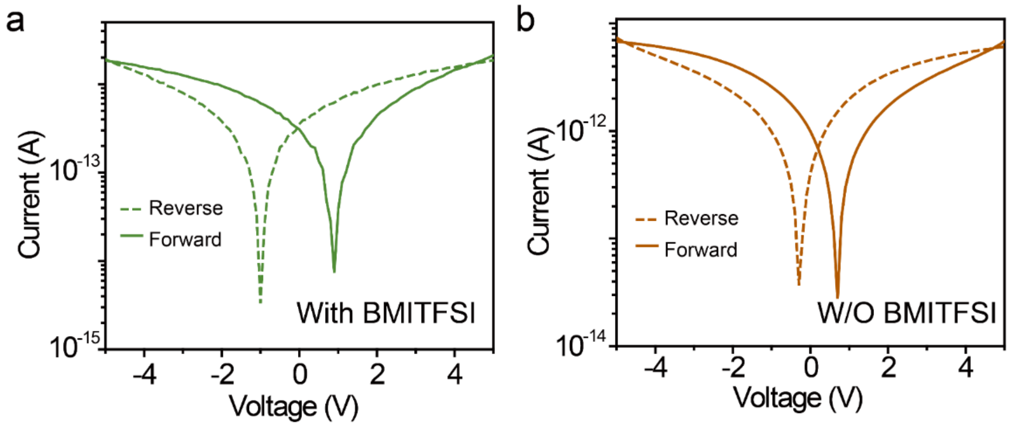

Supplementary Fig. 11. Steady-state *I-V* curves of perovskite films (a) with additive and (b) without additive. The larger hysteresis phenomenon suggests faster ion mobility of perovskite films with additives.

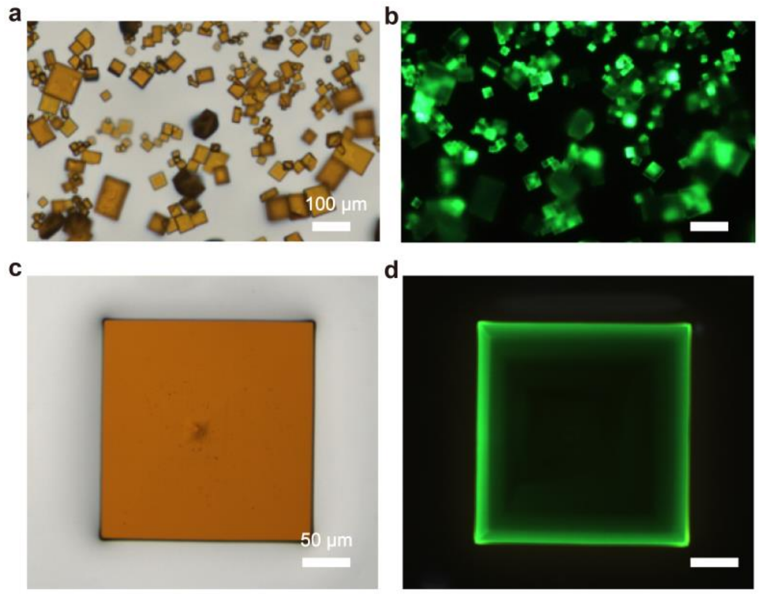

Supplementary Fig. 12. (a, c) Morphology and (b, d) photoluminescence photographs of $MAPbBr_3$ perovskite single-crystal fabricated without BMITFSI additives.

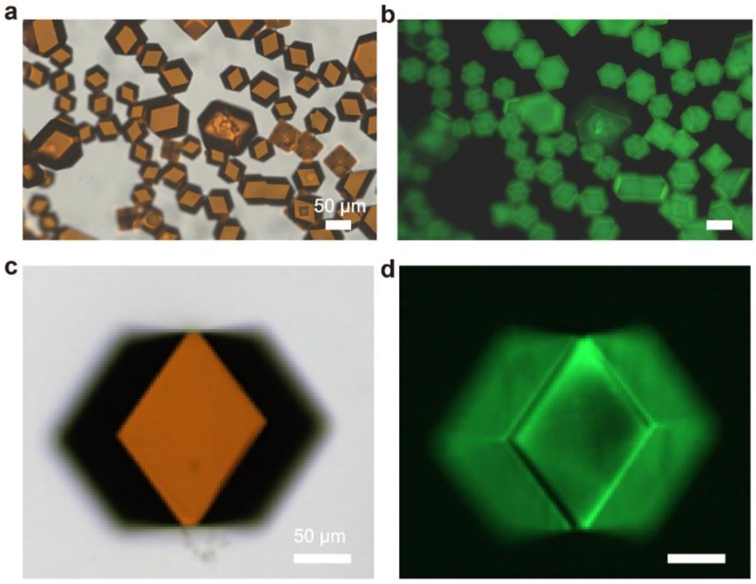

Supplementary Fig. 13. (a, c) Morphology and (b, d) photoluminescence photographs of $MAPbBr_3$ perovskite single-crystal fabricated with BMITFSI additives.

Supplementary Note 1. The fabrication of $MAPbBr_3$ perovskite single-crystal with/without BMITFSI additives.

The perovskite precursor solution (1.5M) was prepared by dissolving $MAPbBr_3$ perovskite powder into a solution of DMF. For the perovskite single-crystal without the BMITFSI additive, single crystals were fabricated by dropping a precursor solution onto a glass substrate. Single crystal microplates were formed after evaporation at room temperature. For a solution of perovskite precursor doped with 20% BMITFSI additive (volume ratio relative to perovskite solution), single crystals were fabricated by dropping the precursor solution onto a glass substrate. Polyhedral single crystals were formed after evaporation at room temperature.

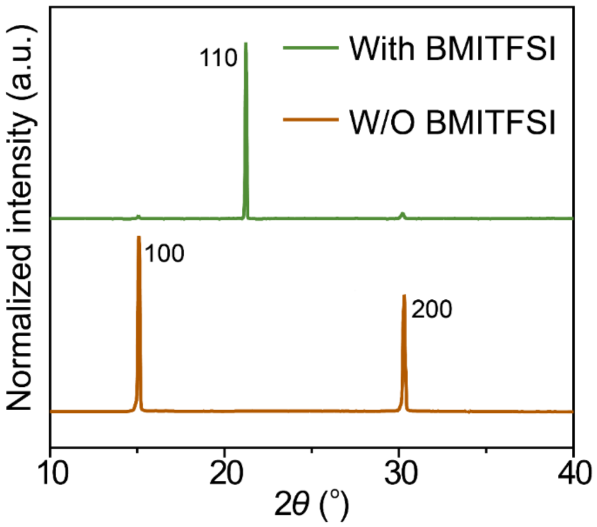

Supplementary Fig. 14. XRD pattern of $MAPbBr_3$ perovskite single-crystal with/without BMITFSI additives, revealing effective modulation of crystal plane orientation.

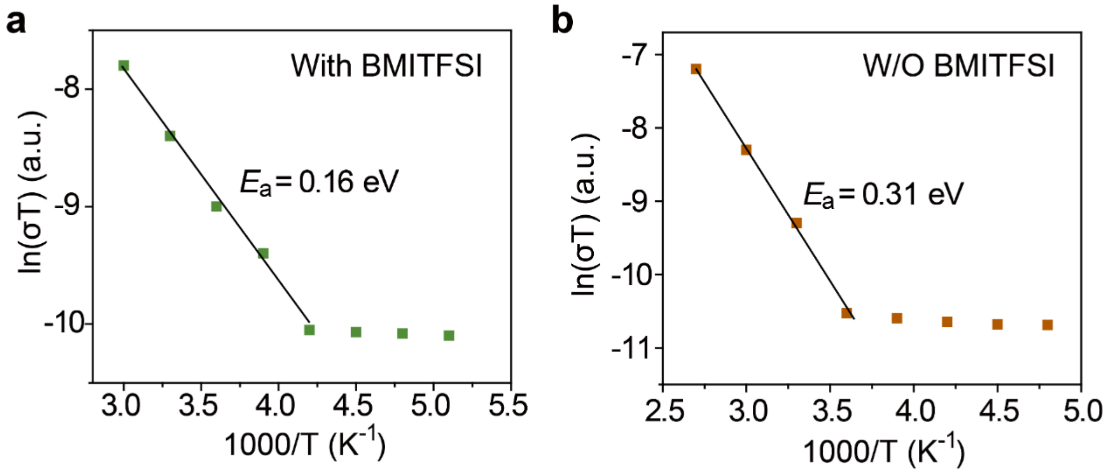

Supplementary Fig. 15. Arrhenius plots of the conductivity for (a) (110) crystal facet with BMITFSI additive, (b) (100) crystal facet without BMITFSI additive devices under dark conditions, where the E_a is calculated from the slope of the curves.

Supplementary Note 2. The calculation of the ion migration activation energy of $MAPbBr_3$ perovskite single-crystal with/without BMITFSI additives.

We then systematically measured the ion migration activation energy of perovskite single-crystal with different crystal facets by temperature-dependent conductivity measurements. The device is based on a lateral photoconductive structure of Au/perovskite/Au. The ion migration activation energy (E_a) can be extracted by fitting the raw data points with the Nernst–Einstein equation,

$$\sigma (T) = \frac{\sigma_0}{T} \exp \left(\frac{-E_a}{k_b T}\right)$$

where k_b is the Boltzmann constant, T is the temperature, and σ_0 is a constant[1, 2]. The ion migration activation energy Ea corresponds to the slope of the ln (σT) - 1000/T relation. At higher temperature regions, Ea values were extracted from the slope of the fitted line. Supplementary Fig. 15. shows Ea values of the (110) crystal facet and the (100) crystal facet calculated by fitting the corresponding Arrhenius plots. Compared to the Ea value of 0.31 eV of the (100) crystal facet, a lower Ea value of 0.16 eV of the (110) crystal facet was demonstrated.

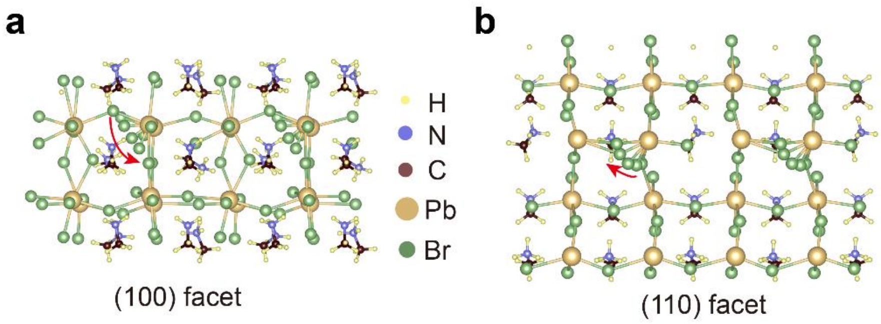

Supplementary Fig. 16. The calculated ion migration path for (a) (100) crystal facets and (b) (110) crystal facets.

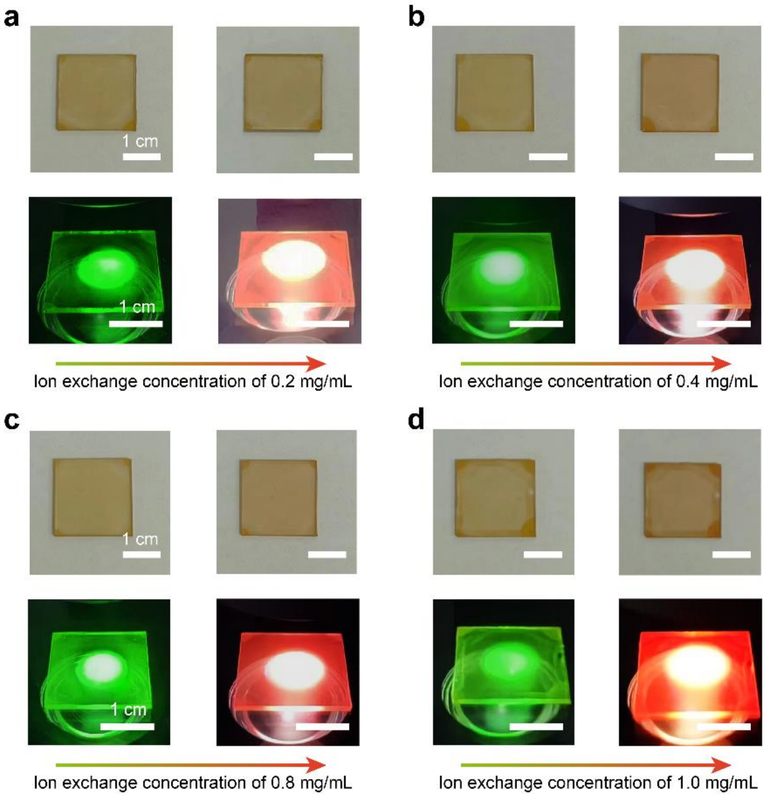

Supplementary Fig. 17. Optical and photoluminescence photographs of perovskite films with additive before and after the ion exchange with MAI concentration of (a) 0.2 mg/ml, (b) 0.4 mg/ml, (c) 0.8 mg/ml, and (d) 1.0 mg/ml.

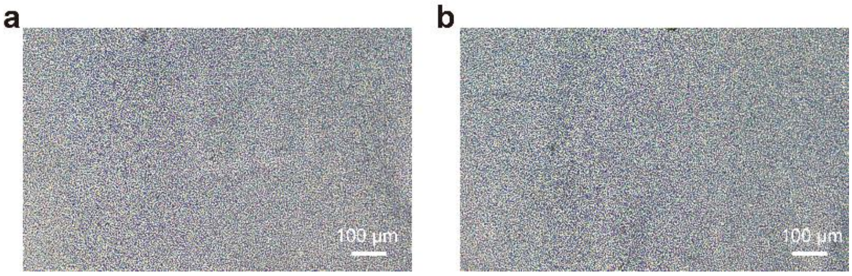

Supplementary Fig. 18. Morphology of perovskite films with additive before and after the ion exchange with MAI concentration of 0.1 mg/ml, indicating that the ion exchange process is a mild and non-destructive process.

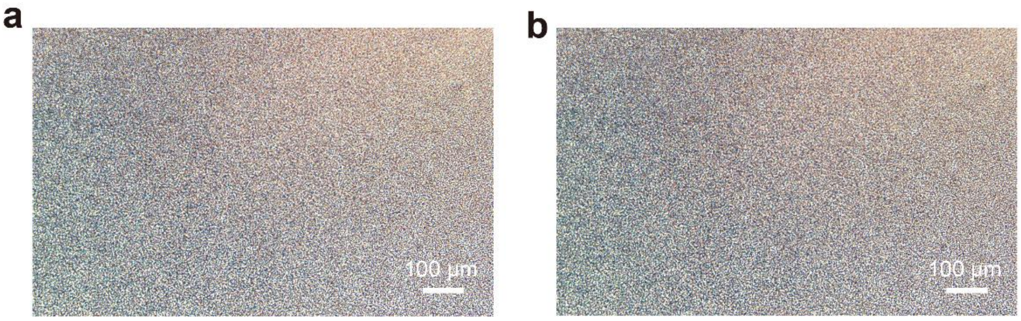

Supplementary Fig. 19. Morphology of perovskite films with additive (a) before and (b) after UV illumination indicates that the ion migration is a mild and non-destructive process.

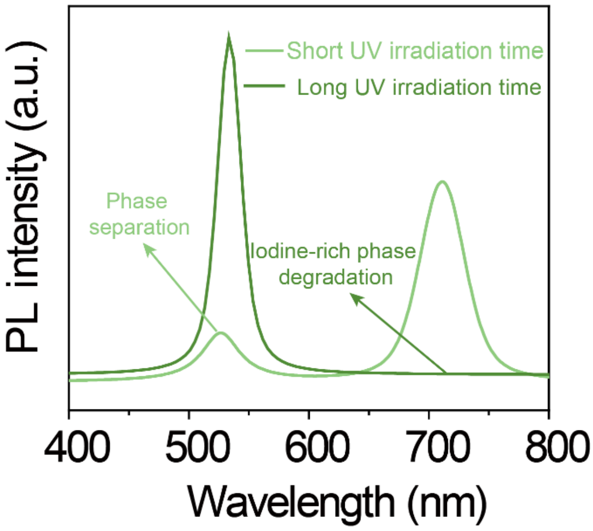

Supplementary Fig. 20. Photoluminescence spectra of the mix-halide perovskite films with MAI concentration of 0.1 mg/ml at different UV irradiation times.

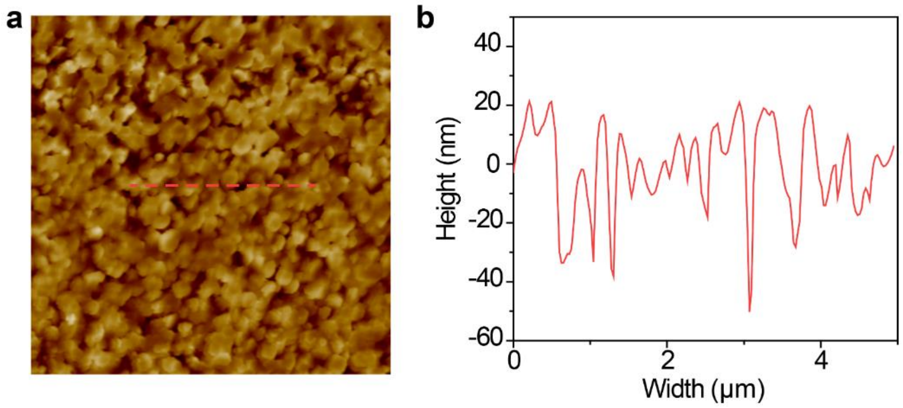

Supplementary Fig. 21. Atomic force microscope (AFM) (a) topography image and (b) the height diagrams of spin-coated binary mix-halide perovskite films, revealing the small grains size of roughly hundred nanometers.

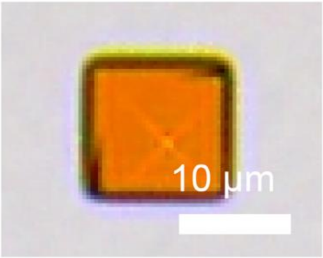

Supplementary Fig. 22. Bright-field photographs of mix-halide perovskite single crystal with a size of roughly 10 micrometers.

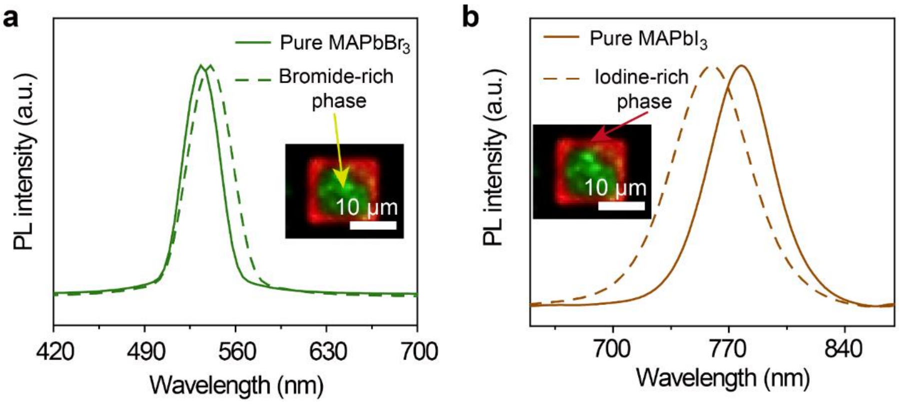

Supplementary Fig. 23. (a) Photoluminescence spectrum of the bromide-rich phase within mix-halide perovskite single crystal and pure $MAPbBr_3$ perovskites, showing a slight PL peak difference. (b) Photoluminescence spectrum of the iodine-rich phase within mix-halide perovskite single crystal and pure $MAPbI_3$ perovskites, showing a small PL peak difference. The small PL peak difference indicates that the composition of the bromide-rich phase and iodine-rich phase is close to the pure $MAPbBr_3$ and $MAPbI_3$ perovskites[3, 4]. Inside images are the corresponding photoluminescence photographs of phase-separated crystals.

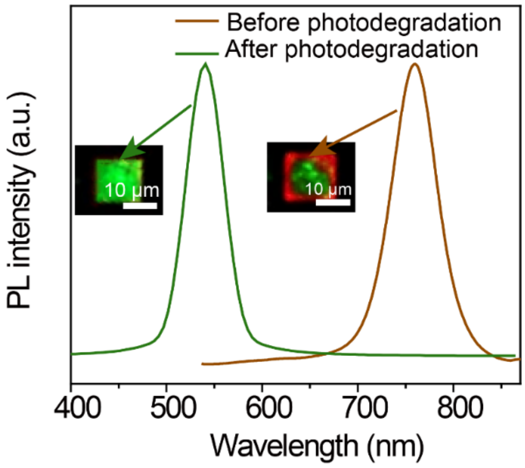

Supplementary Fig. 24. Photoluminescence spectrum of the iodine-rich region within mix-halide perovskite single crystal before photodecomposition and after photodecomposition. The shift of the photoluminescence peak from 753 nm to 543 nm suggests the degradation of the iodine-rich phase. Inside images are the corresponding photoluminescence photographs of phase-separated crystals.

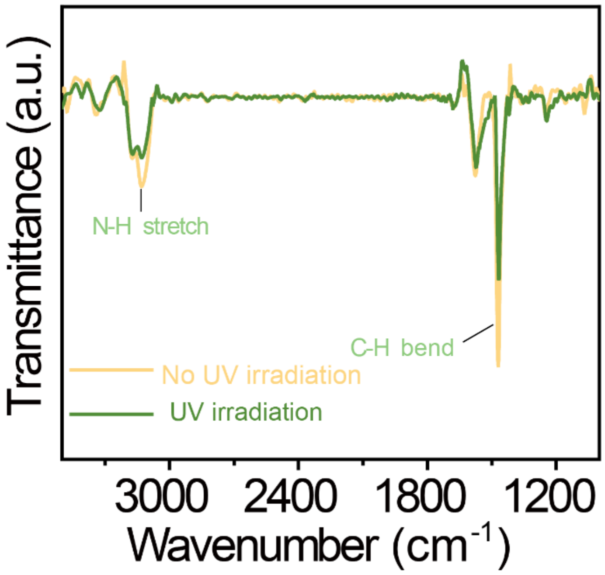

Supplementary Fig. 25. FTIR spectra of mix-halide perovskite films before and after UV irradiation.

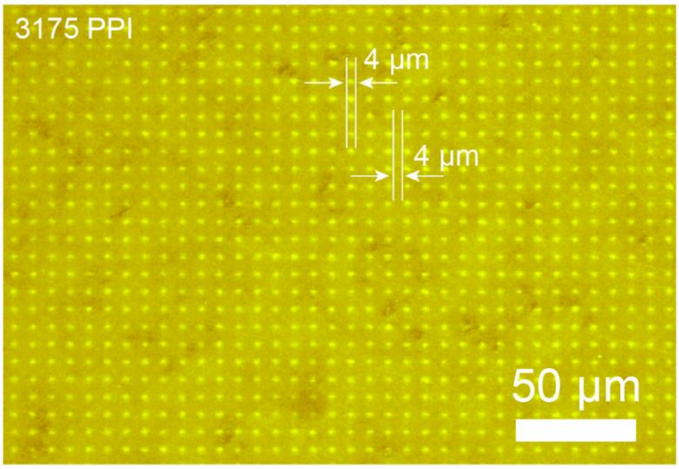

Supplementary Fig. 26. Photoluminescence photographs of photonic pattern information based on direct photo-patterning technique, showing the smallest pattern size of 4 μm, corresponding to a resolution of roughly 3175 PPI.

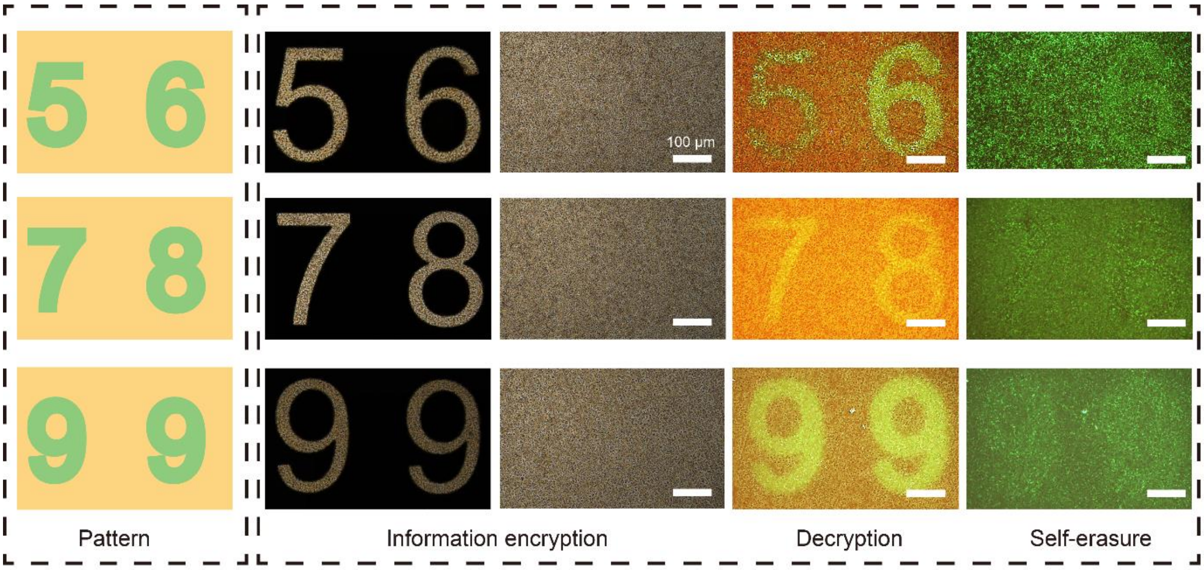

Supplementary Fig. 27. Encryption, decryption, and self-erasure process of photonic pattern information with different numerical patterns from 5 to 9.

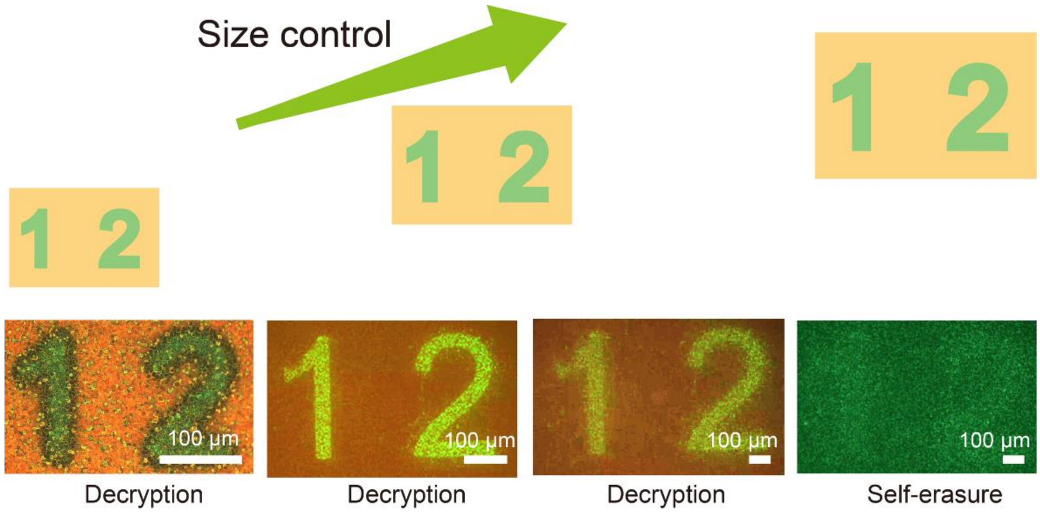

Supplementary Fig. 28. Decryption and self-erasure process of photonic pattern information with different sizes.

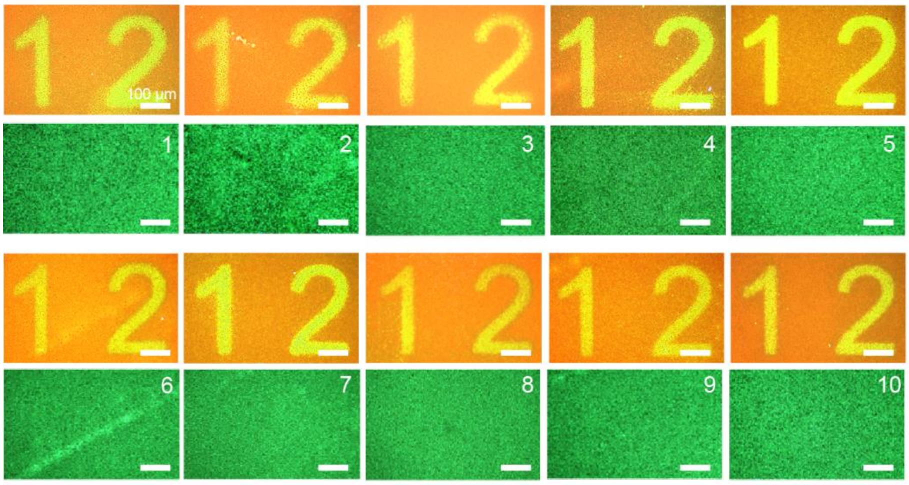

Supplementary Fig. 29. The pattern encryption and erasure process for 10 different photonic cryptography chips, confirming excellent controllability and repeatability. The white number represents different photonic cryptography chips.

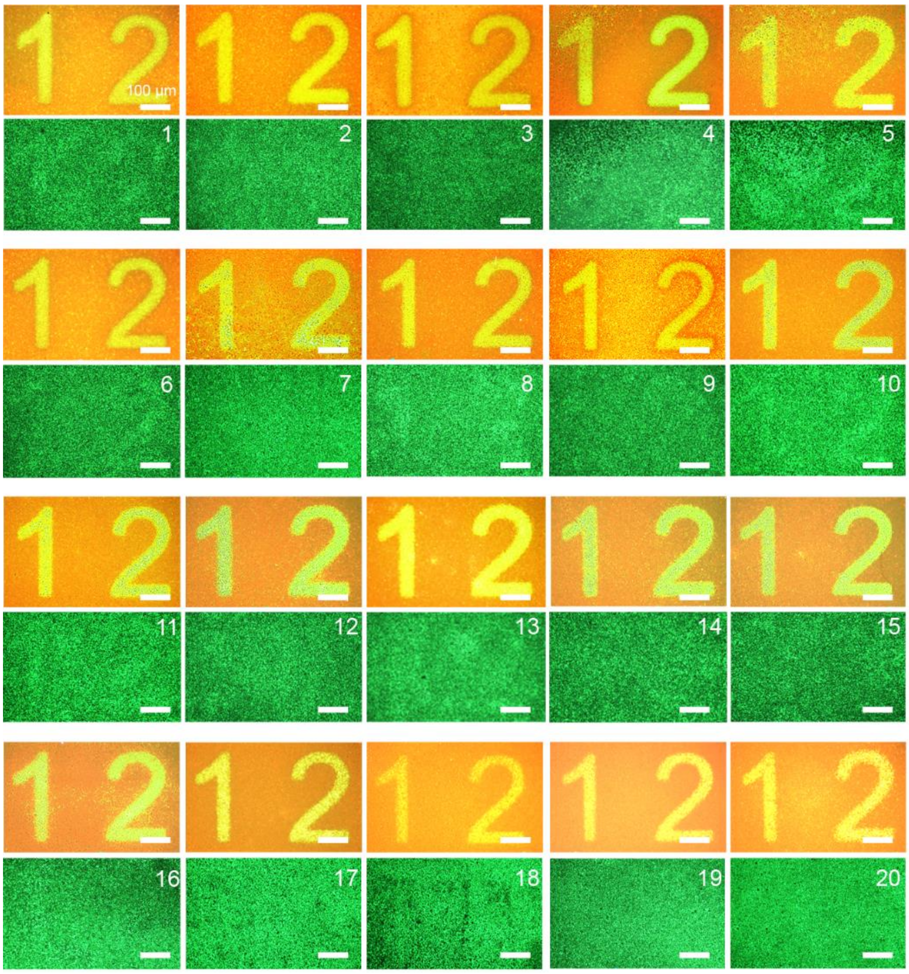

Supplementary Fig. 30. Multiple re-encryption of photonic pattern information, confirming excellent reliability. The white numbers represent the cycle times.

Supplementary Note 3. The detailed reproducible encryption process.

For the fabrication of binary mix-halide perovskite films, MAI of 0.1mg/ml concentration dissolved in an isopropanol solution was spin-coated onto the surface of the $MAPbBr_3$ perovskite film for an ion-exchange reaction. After the first encryption and erasure, pure isopropanol solution was again spin-coated onto the surface of the binary mix-halide perovskite films, thus causing a redistribution of halide ions to compensate for the loss of ions in the previously exposed regions, and then the second encryption and erasure was performed. The multiple repetitive encryption process is consistent with the above process for reproducible encryption. In particular, since a small amount of iodide ions is taken away during the spin-coating of pure isopropanol solution, thus we will again spin-coat MAI of 0.1 mg/ml concentration onto the surface of the binary mix-halide perovskite films after the fifth cycle to replenish the loss of ions.

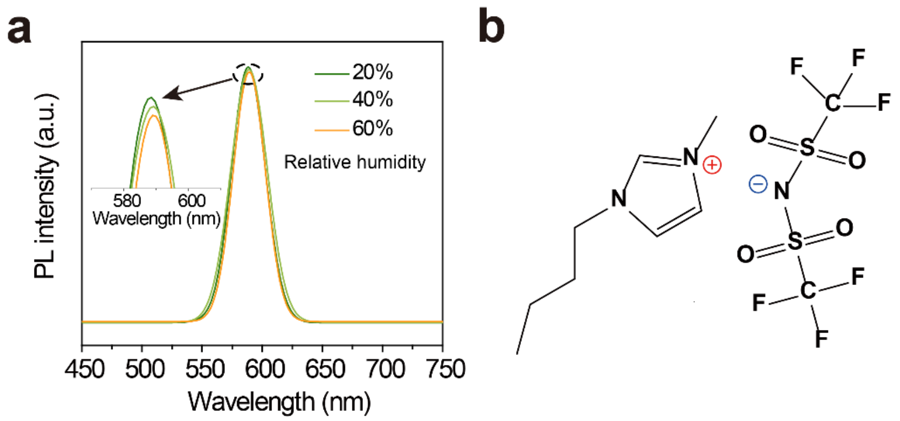

Supplementary Fig. 31. (a) The photoluminescence spectrum of the mix-halide perovskite films under different humidity for 10h (temperature: 25 – 30 ° C, relative humidity: 20-60%). (b) Molecular structure of BMITFSI additives. The photoluminescence spectrum of the mix-halide perovskite films present a slight reduction under different humidity for 10h, indicating good humidity stability of perovskite films, which can be attributed to the hydrophobic effect of the BMITFSI additive.

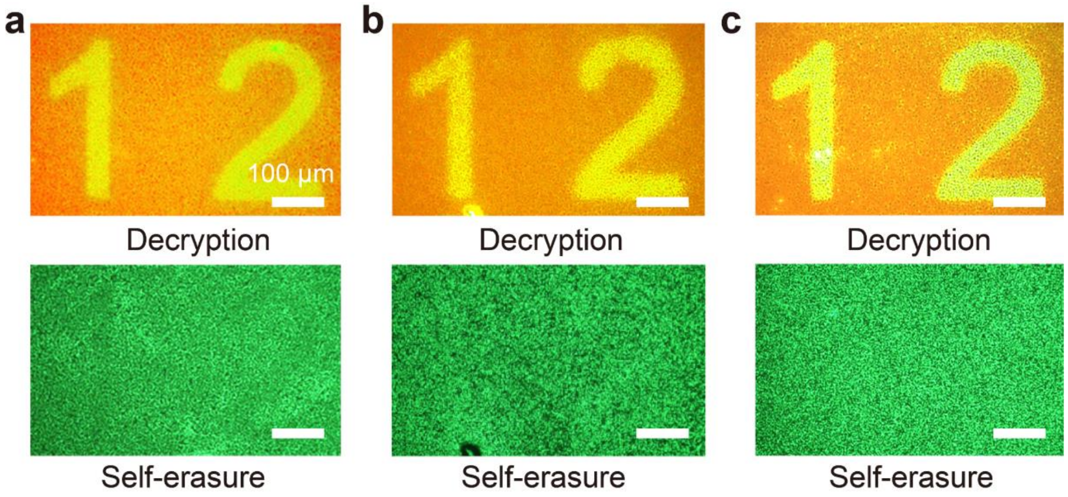

Supplementary Fig. 32. The recording and erasing process of encryption patterns based on the mix-halide perovskite films under different humidity of (a) 20%, (b) 40%, and (c) 60% (temperature: 25–30 °C, relative humidity: 20-60%). The fast encryption and decoding of pattern information significantly reduces the exposure time to moisture in the air and increases the lifetime of the photonic cryptographic chip.

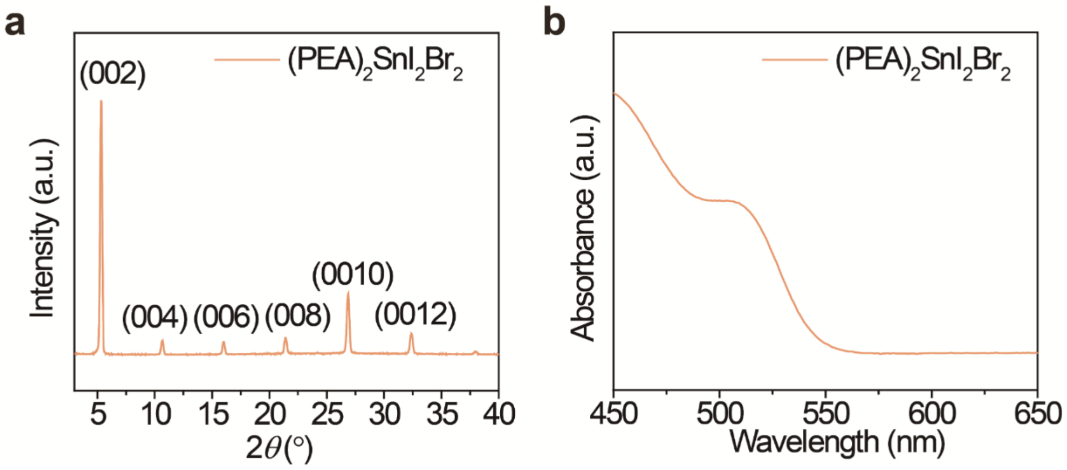

Supplementary Fig. 33. (a) The XRD pattern and (b) absorption spectrum of binary mix-halide $PEA_2SnI_2Br_2$ perovskite films.

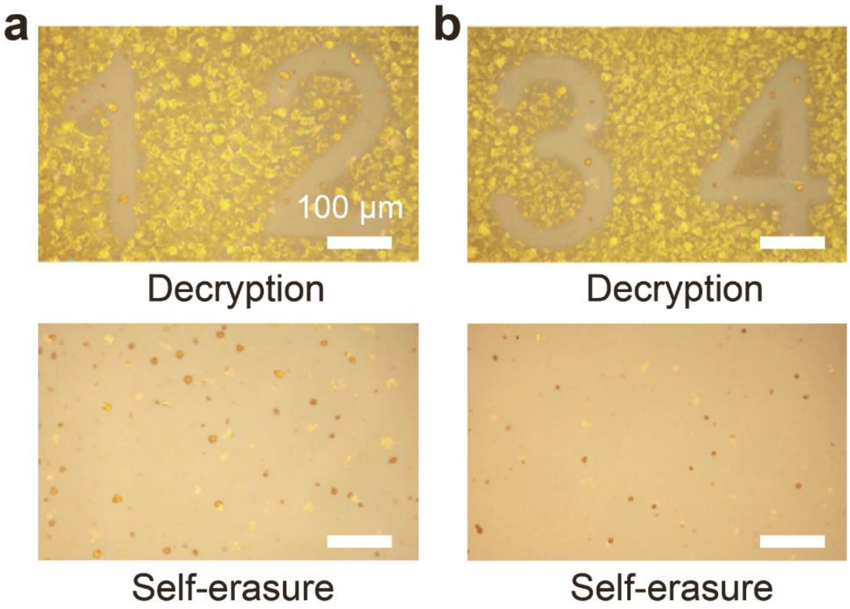

Supplementary Fig. 34. The recording and erasing process of encryption patterns based on the binary mix-halide $PEA_2SnI_2Br_2$ perovskite films.

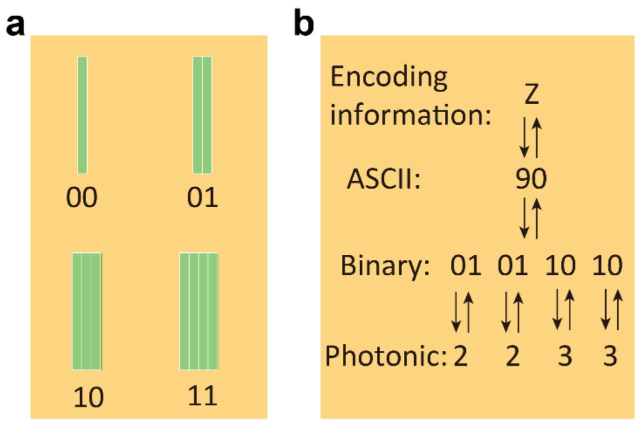

Supplementary Fig. 35. (a) Binary coding rules based on photonic patterns with specific width and length. (b) Conversion mechanism of photonic coding to other coding rules.

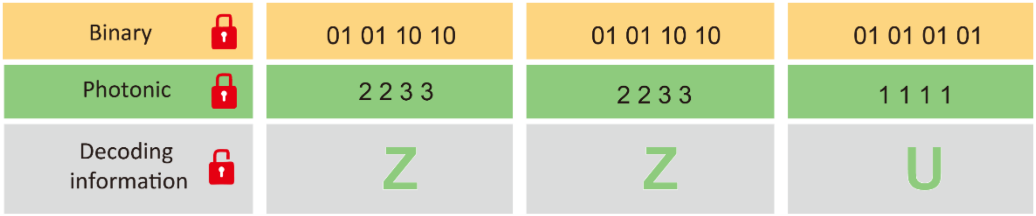

Supplementary Fig. 36. The decoding process of encoding information for “ZZU”.

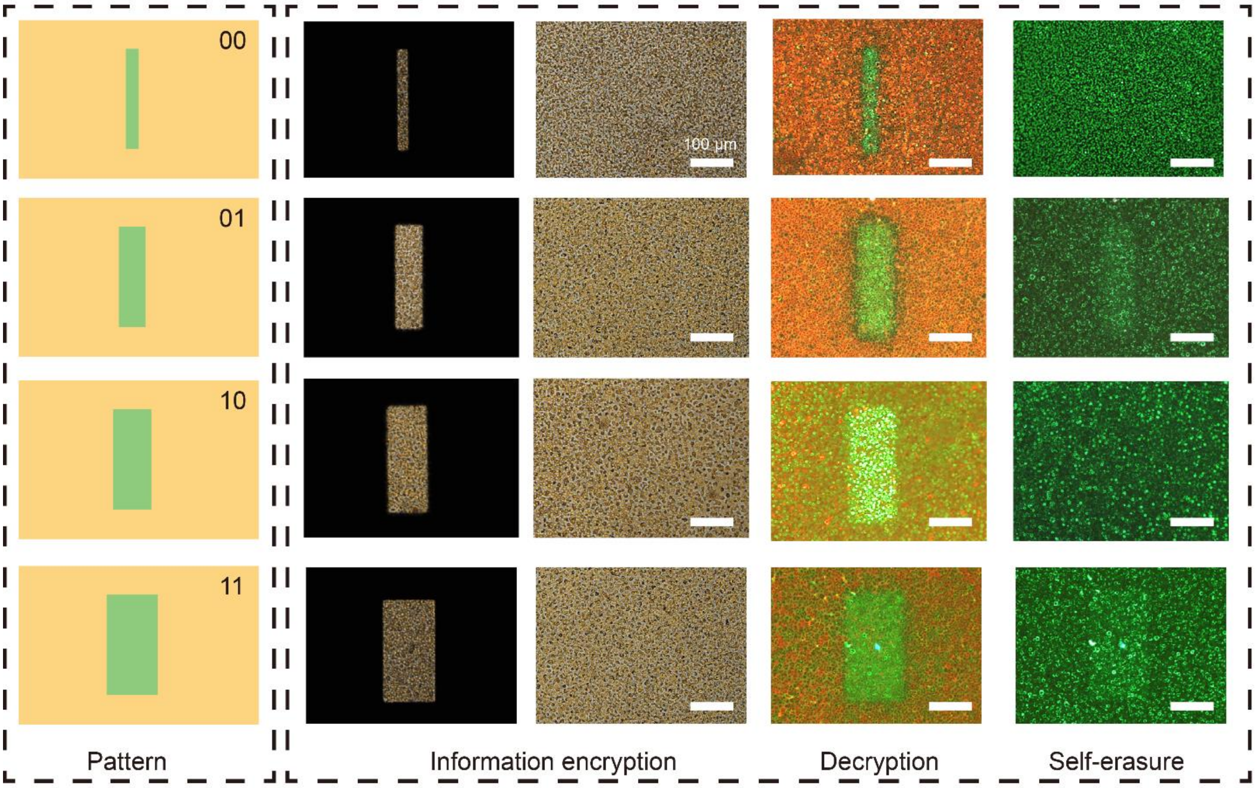

Supplementary Fig. 37. Encryption, decryption, and self-erasure process of photonic encoding information.

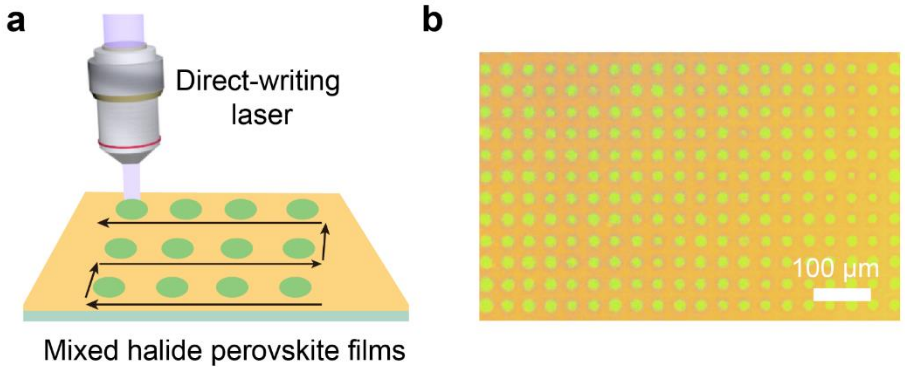

Supplementary Fig. 38. (a) Schematic of the direct-writing lithography technique. (b) Photoluminescence photographs of photonic pattern information based on direct-writing lithography technique.

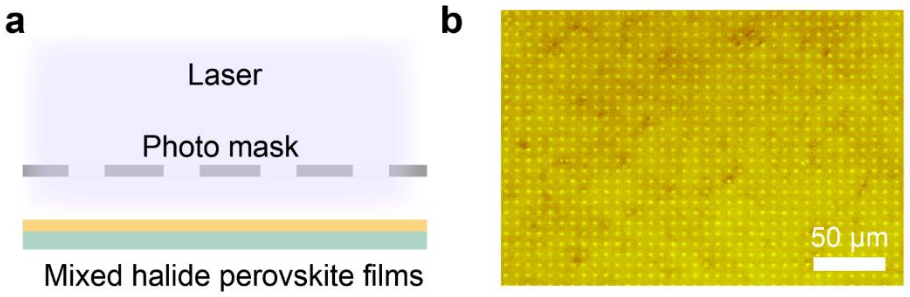

Supplementary Fig. 39. (a) Schematic of the photolithography technique. (b) Photoluminescence photographs of photonic pattern information based on the photolithography technique.

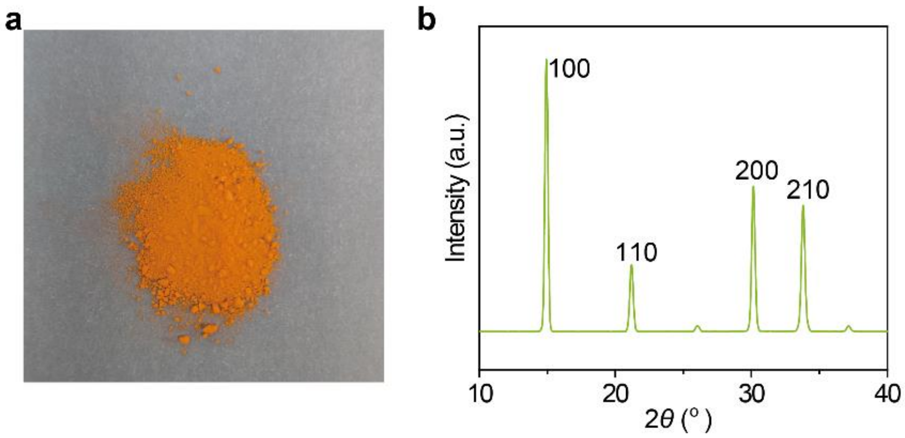

Supplementary Fig. 40. Photographs of (a) $MAPbBr_3$ perovskite powder and (b) XRD result.

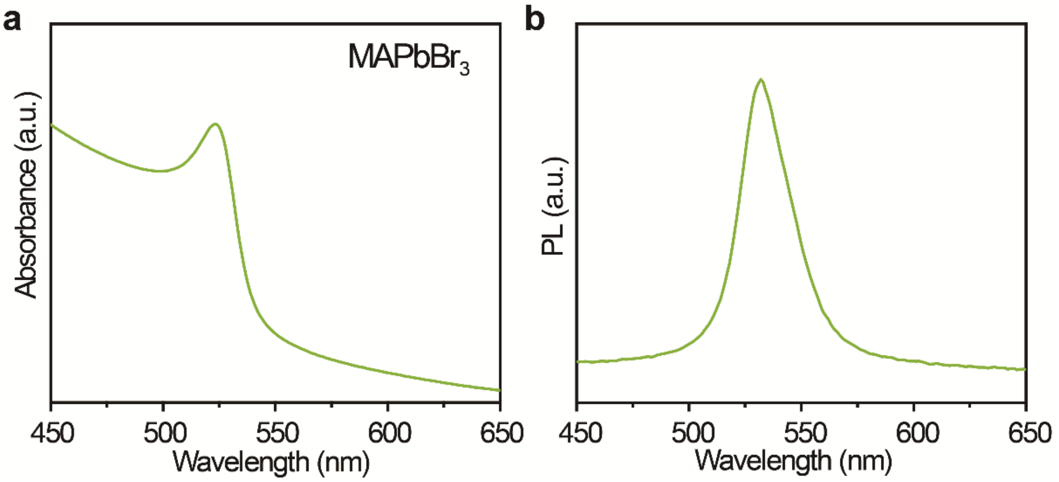

Supplementary Fig. 41. (a) The absorption and (b) photoluminescence spectrum of the $MAPbBr_3$ perovskite film.

Supplementary Table 1. Information coding rules

Control Character	Decimal	Binary	Photonic Code	Control Character	Decimal	Binary	Photonic Code
NUT	0	00000000	1111	$	36	00100100	1321
SOH	1	00000001	1112	%	37	00100101	1322
STX	2	00000010	1113	&	38	00100110	1323
ETX	3	00000011	1114	'	39	00100111	1324
EOT	4	00000100	1121	(	40	00101000	1331
ENQ	5	00000101	1122	)	41	00101001	1332
ACK	6	00000110	1123	*	42	00101010	1333
BEL	7	00000111	1124	+	43	00101011	1334
BS	8	00001000	1131	,	44	00101100	1341
HT	9	00001001	1132	-	45	00101101	1342
LF	10	00001010	1133	.	46	00101110	1343
VT	11	00001011	1134	/	47	00101111	1344
FF	12	00001100	1141	0	48	00110000	1411
CR	13	00001101	1142	1	49	00110001	1412
SO	14	00001110	1143	2	50	00110010	1413
SI	15	00001111	1144	3	51	00110011	1414
DLE	16	00010000	1211	4	52	00110100	1421
DCI	17	00010001	1212	5	53	00110101	1422
DC2	18	00010010	1213	6	54	00110110	1423
DC3	19	00010011	1214	7	55	00110111	1424
DC4	20	00010100	1221	8	56	00111000	1431
NAK	21	00010101	1222	9	57	00111001	1432
SYN	22	00010110	1223	:	58	00111010	1433
TB	23	00010111	1224	;	59	00111011	1434
CAN	24	00011000	1231	<	60	00111100	1441
EM	25	00011001	1232	=	61	00111101	1442
SUB	26	00011010	1233	>	62	00111110	1443
ESC	27	00011011	1234	?	63	00111111	1444
FS	28	00011100	1241	@	64	01000000	2111
GS	29	00011101	1242	A	65	01000001	2112
RS	30	00011110	1243	B	66	01000010	2113
US	31	00011111	1244	C	67	01000011	2114
(space)	32	00100000	1311	D	68	01000100	2121
!	33	00100001	1312	E	69	01000101	2122
"	34	00100010	1313	F	70	01000110	2123
#	35	00100011	1314	G	71	01000111	2124

Supplementary Table 2. Information coding rules

Control Character	Decimal	Binary	Photonic Code	Control Character	Decimal	Binary	Photonic Code
H	72	01001000	2131	m	109	01101101	2342
I	73	01001001	2132	n	110	01101110	2343
J	74	01001010	2133	o	111	01101111	2344
K	75	01001011	2134	p	112	01110000	2411
L	76	01001100	2141	q	113	01110001	2412
M	77	01001101	2142	r	114	01110010	2413
N	78	01001110	2143	s	115	01110011	2414
O	79	01001111	2144	t	116	01110100	2421
P	80	01010000	2211	u	117	01110101	2422
Q	81	01010001	2212	v	118	01110110	2423
R	82	01010010	2213	w	119	01110111	2424
S	83	01010011	2214	x	120	01111000	2431
T	84	01010100	2221	y	121	01111001	2432
U	85	01010101	2222	z	122	01111010	2433
V	86	01010110	2223				
W	87	01010111	2224				
X	88	01011000	2231				
Y	89	01011001	2232				
Z	90	01011010	2233				
[	91	01011011	2234				
\	92	01011100	2241				
]	93	01011101	2242				
^	94	01011110	2243				
_	95	01011111	2244				
`	96	01100000	2311				
a	97	01100001	2312				
b	98	01100010	2313				
c	99	01100011	2314				
d	100	01100100	2321				
e	101	01100101	2322				
f	102	01100110	2323				
g	103	01100111	2324				
h	104	01101000	2331				
i	105	01101001	2332				
j	106	01101010	2333				
k	107	01101011	2334				
l	108	01101100	2341				

Supplementary References

1. Zhao, Y. et al. Suppressing ion migration in metal halide perovskite via interstitial doping with a trace amount of multivalent cations. *Nat. Mater.* **21**, 1396-1402 (2022).

2. Li, N., Jia, Y., Guo, Y. & Zhao, N. Ion Migration in Perovskite Light-Emitting Diodes: Mechanism, Characterizations, and Material and Device Engineering. *Adv. Mater.* **34**, 2108102 (2022).

3. Yoon, S.J. et al. Tracking Iodide and Bromide Ion Segregation in Mixed Halide Lead Perovskites during Photoirradiation. *ACS Energy Lett*. **1**, 290-296 (2016).

4. Liu, Y. et al. 120 mm single-crystalline perovskite and wafers: towards viable applications. *Sci. China Chem*. **60**, 1367-1376 (2017).
