# Supplementary material for: Direct photo-patterning of halide perovskites toward machine-learning-assisted erasable photonic cryptography
Source: Nat Commun. 2025 Apr 7;16:3316. doi: 10.1038/s41467-025-58677-7 (PMC11977006; doi:10.1038/s41467-025-58677-7)
Supplement: Supplementary file 2 — Description of Additional Supplementary Files [file 41467_2025_58677_MOESM2_ESM.pdf]

Description of Additional Supplementary Files

File Name: Supplementary Data 1
Description: The corresponding Computational Data after ion migration for 100 crystal facets.

File Name: Supplementary Data 2
Description: The corresponding Computational Data before ion migration for 100 crystal facets.

File Name: Supplementary Data 3
Description: The corresponding Computational Data after ion migration for 110 crystal facets.

File Name: Supplementary Data 4
Description: The corresponding Computational Data before ion migration for 110 crystal facets.
